# Effects of starch sources varying in particle sizes on ruminal fermentation, nutrient flow, starch digestibility, and lactation performance of dairy cows

**DOI:** 10.1093/jas/skad147

**Published:** 2023-05-11

**Authors:** Maria N T Shipandeni, Eduardo M Paula, Giulia Esposito, Antonio P Faciola, Emiliano Raffrenato

**Affiliations:** Department of Animal Sciences, Faculty of AgriSciences, Stellenbosch University, Private Bag X1, Matieland 7602, South Africa; Department of Animal Production, Agribusiness and Economics, Faculty of Agriculture, Engineering and Natural Sciences, University of Namibia, Private Bag 13301, Windhoek, Namibia; Institute of Animal Science, Beef Cattle Research Center, Sertãozinho, SP 14160-970, Brazil; Department of Veterinary Science, University of Parma, Via Taglio 8, 43126 Parma, Italy; Department of Animal Sciences, University of Florida, Gainesville, FL 32608, USA; Department of Animal Sciences, Faculty of AgriSciences, Stellenbosch University, Private Bag X1, Matieland 7602, South Africa; Department of Comparative Biomedicine and Food Science (BCA), University of Padova, Legnaro 35020, Italy

**Keywords:** grain processing, ruminal digestibility, starch fermentability, starch flow

## Abstract

Cereal grains are the predominant starch source (**SS**) for dairy cows; however, starch digestibility varies greatly depending on source, grain processing, and potentially interactions between these factors. The objective was to study the effects of the interactions between SS, and particle sizes (**PS**) on ruminal fermentation, nutrient flow, starch digestibility, and lactation performance of dairy cows. Four ruminally cannulated multiparous Holstein cows were used in a 4 × 4 Latin square design with a 2 × 2 factorial arrangement of treatments. Two SS (corn or sorghum) used in this study were either finely or coarsely ground (using a 1- or 4-mm screen sieve). Digesta flow was quantified using the reticular sampling technique, applying the triple-marker method. Data were analyzed using the GLIMMIX procedure of SAS version 9.3 (SAS Institute Inc., Cary, NC, USA). For ruminal pH, data were analysed with time as repeated measure. There were no interactions between SS and PS on production or intake, flow, and digestibility of nutrients. Dry matter intake was greater for the corn diet compared to the sorghum diet (25.15 vs. 21.98 kg/d), which consequently affected nutrient intake, however, PS did not affect intake. Milk yield was not affected by SS; however, it was greater for cows fed fine grains than cows fed coarser grains (25.32 vs. 23.16 kg/d). Milk fat and milk protein were not affected by SS or PS. Interactions (SS × PS) were observed for ruminal pH, reticular pH, and volatile fatty acids (**VFA**) concentrations but not for ruminal NH_3_–N concentration. Ruminal and reticular pH were greater for sorghum when coarsely ground and the total VFA concentration was decreased, compared to coarse corn and fine sorghum; however, coarsely grinding corn did not affect ruminal or reticular pH nor VFA concentration. Acetate concentration was lower for corn when finely ground; however, finely grinding sorghum did not affect acetate. Decreasing PS increased ruminal digestibility of starch (87.18% vs. 83.43%), reduced the flow of starch to the reticulum (0.79 vs. 0.96 kg/d) but decreased neutral detergent fiber digestibility in the rumen (30.23% vs. 34.88%). Although SS were differently affected by processing, the effects of PS on production, intake, flow, and digestibility of nutrients were observed regardless of the SS. Furthermore, the effects of decreasing PS on pH and VFA concentrations were more pronounced in sorghum compared to corn.

## Teaser

Decreasing grain particle size increased ruminal digestibility, reducing starch flow to the reticulum but also decreased ruminal neutral detergent fiber degradability. Reducing grain particle size improves starch utilization regardless of the starch source (sorghum or corn) for lactation dairy cows.

## Introduction

Corn and sorghum are the two major sources of starch used in dairy rations, due to great availability and high starch content (Ferraretto et al., 2013; Adesogan et al., 2019). The fate of dietary starch in the rumen and postruminally in dairy cows is highly variable, ranging from less than 30% to greater than 90% ruminal starch digestibility (Moharrery et al., 2014). The digestibility of starch depends on the ­interrelations among intrinsic factors, primarily particle size, grain/endosperm type and processing, and extrinsic factors, such as amount of fiber in the diet and animal factors (e.g., starch intake; Patton et al., 2012; Ferraretto et al., 2013; Mills et al., 2017). Altering the dietary concentration and ruminal fermentability of starch impact the performance of dairy cows by affecting energy intake, partitioning, and absorbed protein (Allen, 2000; Allen et al., 2009). Consequently, rumen starch fermentability has implications on starch utilization and milk production (Bradford and Allen, 2004, 2007; Allen et al., 2009). During processing, starch sources (**SS**) result in different particle sizes (**PS**) distributions and geometric mean particle sizes (Al-Rabadi et al., 2012), which can affect starch digestibility (Ferraretto et al., 2013; Gallo et al., 2016).

The concept of partially shifting the site of starch digestion to the small intestine has been proposed to overcome the negative effects associated with excessive ruminal starch fermentation such as ruminal acidosis, reduced fiber fermentation, and lower efficiency of microbial protein production (Firkins et al., 2001; Allen et al., 2009; Larsen et al., 2009). Moreover, shifting the site of starch digestion from the rumen to the small intestine can potentially provide energetic advantages (Owens et al., 1998; Harmon and Mcleod, 2001; Huntington et al., 2006). However, the review by Trotta et al. (2022) indicates that the absorption of glucose in the small intestine of ruminants is limited.

The site of starch digestion can be modulated by differing degrees of grain processing (Callison et al., 2001, Rémond et al., 2004; Larsen et al., 2009), chemical treatment (Deckardt et al., 2013), or by feeding different grain sources (Herrera-Saldana et al., 1990; McAllister et al., 1990; Taylor and Allen, 2005). Previous literature has indicated inconsistencies regarding corn starch digestion and particle size (Nocek and Tamminga, 1991; Reynolds, 2006). Ferraretto et al. (2013) did not observe effects of PS on dry matter intake (**DMI**) in a meta-analysis, consistent with other studies (Callison et al., 2001; Rémond et al., 2004; Brossillon et al., 2018).

However, Firkins et al. (2001) reported a decrease on DMI and Knowlton et al. (1998) reported an increased DMI associated with changes in particle size. Further inconsistencies around particle size and milk yield have been observed where milk yield was affected by particle size (Knowlton et al., 1996, 1998; Firkins et al., 2001; Rémond et al., 2004) or not (Ferraretto et al., 2013; Fredin et al., 2015; Brossillon et al., 2018). The effects of particle size on milk fat and/or milk protein have also been inconsistent (Knowlton et al., 1996; Ferraretto et al., 2013; Fredin et al., 2015). While the effects of particle size on starch digestibility have been extensively studied in corn, there have been very few studies focused on sorghum particle size; moreover, more research is needed to elucidate the effects of SS and PS interactions on intake, rumen fermentation, nutrient flow, digestibility as well as on milk production.

Additionally, inconsistencies in literature on the interaction of particle size and SS demonstrate a need to compare these interactions on cow performance, feed efficiency, and nutrient utilization. Therefore, the objective of this study was to study the interactions between SS and PS on ruminal fermentation parameters, nutrient flow, starch digestibility, and lactation performance of dairy cows. We hypothesized that SS (corn vs. sorghum) and grain processing (1 vs. 4 mm) may interact in various animal responses.

## Materials and Methods

The experiment was conducted at the Welgevallen Experimental Farm of Stellenbosch University and carried out in accordance with the South African National Standard for the care and use of Animals for Scientific Purpose (SANS 10386: 2008). Animal care and all experimental procedures were approved by the Stellenbosch University’s Research Ethics Committee: Animal Care and Use (REC: ACU) (protocol number: SU-ACUD15-00064).

### Animals, experimental design, and diets

Four multiparous lactating Holstein-Friesian dairy cows (means ± SD: 718 ± 59 kg of BW; 230 ± 57 d in milk, 25.29 ± 6.80 milk yield/d at the beginning of the experiment) fitted with ruminal cannulas (10 cm i.d.; Bar Diamond Inc., Parma, ID) were used in a 4 × 4 Latin square design experiment with a 2 × 2 factorial arrangement of treatments; two SS (corn or sorghum) either finely or coarsely ground (using a 1- or 4-mm screen sieve). The duration of each experimental period was 16 d, with the first 10 d for adaptation and the last 6 d used for data collection. Because of the small differences between treatments, adaptation periods were shorter than what usually has been recommended (Grant et al., 2015). Grant and collaborators (2015) demonstrated that response to diet for eating, ruminating and resting behavior stabilizes within 1 to 7 d, therefore an adaptation period of 7 to 14 d is sufficient for experiments investigating DMI and eating behavior, except for diets with extreme differences in their level of digestibility. We aimed to reduce the adaptation periods in order to minimize confounding effects due to milk yield reduction and physiological changes occurring towards the end of the lactation. Cereal grains were sourced locally, dried at 60 °C for 48 h in a forced-air oven and milled using a hammer mill (Drotsky M16, Aktief (Pty) Ltd, Johannesburg, South Africa) fitted with a 1-mm or a 4-mm screen representing fine and coarse particle sizes, respectively. The size of the sieves used in this experiment were selected based on results from a preliminary in vitro study conducted in our laboratory (Shipandeni et al., 2020).

Prior to the experiment, the PS distributions (% of DM retained on each sieve) and geometric distribution of particle sizes (**GMPS**) of milled corn and sorghum grains was determined by sieving. The GMPS is the most common method used to compare PS distribution of cereal grains (ASABE, 2007). A representative sample (~100 g) was sieved for 20 min through a series of 7 screen sieves with nominal aperture sizes of 125, 250, 500, 850, 1,180, 2,000, and 3,350 µm using a sieve shaker (Kingtest laboratory test sieve, Retsch GmbH, Series AS 200 basic, Haan, Germany) at an amplitude of 100 mm. A bottom pan was included to retain particles less than 125 µm. Each sieve was individually weighed before and after each sieving to obtain the weight of the samples on each sieve, thus determining PS distribution and the geometric mean particle size. Three runs per cereal grain were done, and sieves were cleaned thoroughly with a brush per run. The mean particle size was calculated using a log normal distribution (Baker and Herrman, 2002). The nominal geometric mean particle size (**NGMPS**) was calculated, which is based on the smallest dimension of square openings in sieves. Besides particle size distribution, in vitro fermentation conditions were achieved following the procedure of Goering and Van Soest (1970). After incubations, 7 h in vitro starch degradability was performed as recommended by Sniffen and Ward (2011), to determine the in vitro starch degradability of the milled grains (1 and 4 mm; corn and sorghum). Rumen fluid was collected from two ruminally cannulated Holstein dairy cows before morning feeding and mixed in prewarmed insulated thermos flasks. Cows were fed a total mixed ration consisting of roughage (40%) and a concentrate mixture (60%). Ground grains (~0.5 g) were incubated in 125-mL Erlenmeyer flasks (39.5 °C in a water bath under constant CO_2_ bubbling) including blanks. Rates of starch degradation were computed using the 7 h formula (Sniffen and Ward, 2011) as follows:


$$\begin{array}{l} K_{d}-7=\left(\frac{\begin{array}{l} ((LN(Strach,\;\%DM))-\\ (LN(((100-7h\;ivSd,\%Strach)/100)*\\ Strach,\;\%DM))) \end{array}}{7}\right)\times 100, \end{array}$$

where starch is the starch content of the grain on DM basis and ivSd is the in vitro starch degradability as % of starch. Nutrient composition, in vitro starch degradability and rate of starch degradation for corn and sorghum milled with 1- and 4-mm screen are presented in Table 1.

**Table 1.** Nutrient composition, in vitro starch degradability and rate of starch degradation for corn and sorghum milled with 1- and 4-mm screen

Nutrient composition^1^, % DM	Corn		Sorghum	
	Fine (1 mm)	Coarse (4 mm)	Fine (1 mm)	Coarse (4 mm)
**DM, % of fresh matter**	86.51	86.84	87.43	86.67
**Ash**	0.99	0.78	1.05	1.08
**OM**	99.01	99.22	98.95	98.92
**Starch**	76.53	73.99	72.13	71.40
**CP**	7.73	7.73	12.90	12.90
**EE**	4.00	4.00	3.69	3.69
**NDF**	18.69	18.41	9.71	15.81
**ADF**	2.63	4.20	4.35	6.94
**Starch degradability 7h** ^2^ **, %**	78.14	63.51	68.30	60.06
**k**_**d**_ ^3^ **, % h** ^**-1**^	22.52	16.84	14.41	13.11

^1^DM = dry matter; OM = organic matter; CP = crude protein; EE = ether extract; NDF = neutral detergent fiber; ADF, acid detergent fiber.

^2^Starch degradability measured in vitro after 7-h incubation.

^3^k_d_ = rate of starch degradation.

Experimental diets were fed as a total mixed ration (**TMR**) formulated to meet the dietary requirement of lactating dairy cows according to National Research Council (NRC, 2001) and the respective ingredients and nutritional composition are presented in Table 2. Alfalfa was chopped together with wheat straw using a feed mixer (Storti Husky MT 50, Storti S.p.A, Belfiore (VR), Italy) to obtain a theoretical chop length of 4 cm. Experimental diet ingredients (alfalfa + wheat straw, milled grains and high protein concentrate were weighed and mixed in a concrete mixer per feeding time per animal, with addition of water. Cows were individually housed in roofed pens bedded with sand and fed a TMR twice daily (equal portions) at 0730 and 1700 h, after each milking which was done at 0600 and 1600 h. The amounts of TMR offered were adjusted daily throughout the trial depending on the previous day’s intakes to allow for 5% to 10% of refusals. Cows had free access to water troughs during the entire experiment.

**Table 2.** Ingredients and chemical composition (% of DM unless otherwise stated) of the experimental diets containing either corn or sorghum grain ground either finely or coarsely fed as TMR to lactating dairy cows

Item	Treatment			
	Corn		Sorghum	
	Finely	Coarse	Finely	Coarse
Alfalfa hay	41.5	41.5	41.5	41.5
Wheat straw	1.9	1.9	1.9	1.9
Corn	37.7	37.7	_	_
Sorghum	_	_	37.7	37.7
High protein concentrate^1^	18.9	18.9	18.9	18.9
Nutrient composition^2^, % of DM				
DM, % as fed	50.7	50.3	50.6	50.2
OM	93.0	93.0	92.9	92.8
Ash	7.0	7.1	7.1	7.2
Starch	26.3	26.2	25.0	24.7
CP	17.3	17.4	18.9	19.2
EE	2.6	2.7	2.7	2.6
NDF	31.5	32.1	30.8	31.5
uNDF	16.2	16.0	16.0	16.3
ADL	4.3	4.3	4.3	4.4
pdNDF^3^	15.3	16.1	14.7	15.2
NFC^4^	41.7	40.8	40.5	39.5

^1^Sourced from Afgri (Afgri Animal Feeds, Centurion, GP, South Africa), contained Amminomax soya, Gluten 21, Blood meal spray dried, Molasses, Urea, Limestone, Salt, Mineral Premix, Poultry by-product, Monocalcium Phosphate.

^2^DM, dry matter; OM, organic matter; CP, crude protein; EE, ether extract; NDF, neutral detergent fiber; uNDF, undigestible NDF; ADL, acid detergent lignin; pdNDF, potentially digestible; NFC, non-fibrous carbohydrates.

^3^Calculated as NDF − uNDF.

^4^Calculated as 100 − (CP % + NDF % + ether extract % + ash %); NRC, 2001.

### Measurements and sampling procedures

Reticular sampling technique was used as described by Krizsan et al. (2010), using the triple-marker method (France and Siddons, 1986). Markers used were Cobalt ethylenediamine tetra-acetic acid (Co-EDTA), for the fluid/liquid phase, Ytterbium-acetate (Yb-acetate) for the small/solid particle phase, and undigestible neutral detergent fiber (uNDF) for the large particle phase. The Co-EDTA was prepared as described by Udén et al. (1980) and Yb-acetate was obtained from a commercial source (Sigma-Aldrich, Gauteng, South Africa). Undigestible NDF, described below, is naturally present in the ingredients and was used as an internal marker. Briefly, Markers (Co-EDTA [18 g/d] and Yb-acetate [3.78 g/d]) were dissolved in 3-L of distilled water per cow per day during each period. Markers prepared per period were mixed thoroughly to achieve a homogenous marker solution and a sample was collected to determine the actual concentration of markers. The infusion of markers Co-EDTA (2.5 g/d of Co) and Yb-acetate (1.5 g/d of Yb) started on day 11 of each experimental period until the last sampling of digesta on day 16, and reticular sampling was carried out on the last 3 d of the experimental period (days 14 to 16). Each cow received the marker solution (CoEDTA and Yb-acetate) directly into the rumen at the rate of 125 mL/h via cannula fitted with the infusion line (i.d. = 4 mm), using an injector (SimcroTM, New Zealand).

Briefly, 400-mL reticular samples were collected over three consecutive days, four times daily at the interval of 6 h. The sampling times were 0800, 1400, 2000, and 0200 h on day 1, 1000, 1600, 2200, and 0400 h on day 2, and 1200, 1800, 2400, and 0600 h on day 3 of sampling, allowing 12 representative samples to be collected. Briefly, to collect the reticular digesta, a Selekt Cattle Pump and Rumen Fluid Collector (Nimrod Veterinary Products Ltd, Moreton-in-Marsh, Gloucestershire, UK) were inserted into the reticulum through the rumen while holding the steel tip of the pump, which was perforated to prevent rumen digesta from flowing into the pump. While in the reticulum the tip of the pump was released, flushed about 3 to 4 times before collecting 1.0-L of reticular digesta. The reticular digesta pH was measured immediately after sampling using a portable pH meter (Crison PH25 pH meter, Lasec, SA). The 1.0-L of reticular sample of digesta was divided into subsamples as follows: 400 mL reticular digesta for digesta flow analysis and 150 mL for spare, the remaining fluids were discarded. Samples were placed on ice immediately after collection. The 400-mL reticular subsamples from every time point were pooled and held at −20 °C as they were collected to yield a 4.8-L reticular composite samples from each cow in each period. The reticular digesta samples were not filtered/sieved to discard large particles.

Rumen content samples were collected from the ventral area of the rumen before the reticular sampling and rumen pH was measured immediately. The rumen content samples were placed in crushed ice immediately after sampling and stored at −20 °C. Fecal samples were collected (approximately 250 g) on the same 3 d as the reticular and rumen sampling per cow, applying the same sampling time schedule. Fecal samples were collected from the rectum or off ground on a concrete floor immediately after defecation, and not mixed with urine. Samples were stored at −20 °C pending further processing. Fecal samples were used to determine the total tract digestibility using uNDF as internal marker and for marker recoveries.

Milk yield was automatically recorded at each milking using the AfiMilk dairy farming system (AfiMilk Ltd, Kibbutz Afikim, Israel) throughout the experiment. Milk samples were collected during morning and afternoon milking for four consecutive days starting from day 13 to 16 of each period. Daily milk sample (40 mL) composites were obtained by pooling morning and afternoon milk samples based on the proportion of milk yield per cow at each milking. Composite milk samples were preserved with broad spectrum microtabs, stored at room temperature, and delivered to a South African National Accreditation System (SANAS) accredited testing laboratory, MilkoLab (GE Dairy Supplies, Parow, Cape Town, South Africa) for fat, true protein, lactose, somatic cell counts and milk urea nitrogen (**MUN**) by means of infrared analysis (CombiFossTM FT+, Hillerød, Denmark). Body weight (**BW**) was recorded daily during milking throughout the experiment using the AfiWeigh system (AfiMilk Ltd, Kibbutz Afikim, Israel). However, only weight recorded from three consecutive days at the beginning and the end of each period was used to calculate the mean BW of cows for each experimental period. To determine the feed intake, feed offered, and orts were recorded daily per cow before morning feeding for the entire experiment. However, only data from days 11 to 16 were used for data analysis. Samples of TMR and orts were collected daily from days 11 to 16, stored at −20 °C until further analysis.

### Sample preparation

Reticular digesta samples, which were pooled by period for each cow, were thawed at room temperature and separated into three different phases: reticular large particle (**LP**), reticular small particle (**SP**), and reticular fluid (liquid) phase (**FP**) as described by Reynal and Broderick (2005). Briefly, reticular samples were squeezed through one layer of cheesecloth and particles retained on the cheesecloth were defined as the large particle phase. The filtrate was transferred to a 250-mL centrifuge bottle (Nalgene, model 2189-0008, Thermo Fisher Scientific, USA) and centrifuged at 14,000 × *g* (rotor, J14) for 5 min at 4 °C using a Beckman coulter centrifuge (Avanti J-E series, Beckman Coulter, USA). The supernatant was poured off and defined as the fluid phase and the pellet was defined as the small particle phase. The separated phases were stored at −20 °C until lyophilized. The large particle phase was ground to pass through a 1-mm sieve using a Wiley mill (Model 4, Thomas-Scientific, USA), and the small particle and liquid phase was ground using a coffee grinder. The concentration of Co, Yb, and uNDF determined in the LP and SP and of Co and Yb determined in the FP was used to mix DM from freeze-dried FP, SP, and LP in the correct proportions to reconstitute the reticular true digesta (RTD) flowing out of the rumen based on the triple-marker method of France and Siddons (1986). Concentrations of Co, Yb, and uNDF were greater than the other markers in FP, SP, and LP, respectively, allowing for application of the triple-marker approach. The RTD were analysed for DM, ash, starch, crude protein (**CP**), NDF, acid detergent fiber, and uNDF. Pooled rumen contents from each period were thawed at room temperature and subsamples were taken for ruminal volatile fatty acid (**VFA**) and ammonia (NH_3_) analyses. Feed, orts, and fecal samples were also thawed at room temperature, pooled per cow over each sampling period, thoroughly mixed by hands and subsampled. Subsamples were dried in a forced air oven at 60 °C for 72 h to determine DM content and ground to pass through a 1-mm sieve using a Wiley mill (Model 4, Thomas-Scientific, USA) pending analysis.

### Undigestible NDF and chemical analyses

The concentration of uNDF in all TMR samples, digesta fractions (LP, SP, but not the fluid phase; Ahvenjärvi et al., 2000), orts and fecal samples was determined using a long term (240 h) in vitro fermentation as described by Raffrenato et al. (2018). Concentrations of external markers (Cobalt [Co] and Ytterbium [Yb]) in the individual digesta phases, fecal samples and marker samples were analysed using inductively coupled plasma Optical Emission Spectroscopy (ICP-OES/AES) (Thermo iCAP 6000, Thermo Scientific, USA). Briefly, samples (0.3 g) were weighed directly into the Microwave digester Teflon vessels (The Chemours Company,Wilmington, Delaware, USA). Nitric acid (HNO3, 3.5 mL) and hydrogen peroxide (H_2_O_2_, 1 mL) were added to predigest samples for about 15 min before adding deionized water (2.5 mL). Samples were digested using CEM MARS microwave digester at 210 °C and 800 PSI. Vessels were cooled, and samples were diluted (10-fold) to reduce the acid concentration prior to analysis for Co and Yb. Ruminal VFA concentrations were determined using a gas chromatograph-mass spectrometry (GC-MS; Thermo Scientific TriPlus RSH, TRACE 1300, Italy), with clean-up procedure of rumen content samples (modified from Siegfried et al., 1984) which deproteinises the rumen content samples and removes the sugars, and using crotonic acid as the internal standard. The concentrations of NH_3_ in the rumen fluid were analysed using NH_3_ slides on the IDEXX VetTest Chemistry Analyser (IDEXX Laboratories PTY Ltd, South Africa), and a dilution factor of 20.

The DM and ash content were determined according to the AOAC (2002), official Method 934.01 (100 °C; 24 h) and 942.05 (500 °C; 6 h), respectively. The starch content was determined as described by Hall (2009). CP was ­determined by Dumas combustion method (AOAC, 2002; method 992.15) using a LECO nitrogen analyser (model FP-528, Leco Corporation, USA) and ether extract (EE) by using a Tecator Soxtec System HT 1043 Extraction Unit (AOAC, 2002; Method 920.39). NDF was analysed as described by Mertens (2002) using sodium sulphite and thermostable α-amylase, and acid detergent lignin was determined according to Goering and Van Soest. (1970) as modified by Raffrenato and Van Amburgh (2011). Organic matter (**OM**) was calculated as 100 − % ash; nonfiber carbohydrates was calculated as 100 −  (% CP + % aNDFom + % EE + % ash) according to the NRC (2001); and potentially digestible NDF (pdNDF) was calculated as NDF − uNDF. All samples were analysed in duplicate.

### Calculations and statistical analysis

Nutrient intake was calculated using the amounts and compositions of feed offered and refused. Nutrient flow was calculated using the reconstitution system based on the three markers (co-EDTA, Yb, and uNDF) using the procedures described by Krizsan et al. (2010).

Total-tract nutrient digestibilities were calculated from uNDF as an internal marker and nutrient concentration in the refusals-adjusted diet and feces as described by Ferraretto et al. (2015) using the following equation: Apparent total tract nutrient digestibility (% of nutrient intake) = 100 −  [(TMR uNDF/fecal uNDF) × (fecal nutrient content/TMR nutrient content)].

Production data, nutrient flow, ammonia, and volatile fatty acids and digestibility were analysed using the GLIMMIX procedure of SAS (Version 9.3; SAS Institute Inc., Cary, NC, USA) according to a 4 × 4 Latin square design with 2 × 2 factorial arrangements of treatments, with PS and SS, and their interaction, treated as main fixed effects. The random effect of period and cow were also included in the model. For ruminal pH, data were analysed using the GLIMMIX procedure of SAS (Version 9.3; SAS Institute Inc., Cary, NC, USA) with time as a repeated measure, various covariance structures were tested, and the compound symmetry covariance matrix structure was selected because it resulted in the smallest Bayesian and Akaike information criteria. Statistical significance and trends were considered at *P* ≤ 0.05 and 0.05 < *P* ≤ 0.10, respectively. Results are reported as least square means and respective standard errors. Post hoc comparisons were performed using Tukey’s test.

## Results

### Particle distribution, composition, and starch digestibility of milled grains

Although the statistical analysis of PS distributions and NGMPS of SS was not performed because corn and sorghum came from the same source, the distribution of particles among the different sieves was described, but not compared (Table 3). At 1-mm screen size, most particles (80.2% and 80.2% of corn and sorghum, respectively) were below the ≤ 250 µm sieves, with little material (19.8% and 19.7%, respectively) retained in the ≥ 500 µm sieves. In contrast, when the hammer mill screen size was increased to 4 mm, most of the particles were retained in the ≥ 500 µm sieves (60.2% for corn and 76.2% for sorghum), with only 39.8% and 23.8% above the sieves lower than 250 µm for corn and sorghum, respectively. The proportion of particles retained on the smaller sieves (<500 µm) increased with extent of processing of both grains. Reducing hammer mill screen size decreased the NGMPS for both grains (728.54 ± 2.29 vs. 250.62 ± 2.29, means and ± SD, respectively). The starch content of corn was 76.5% and 73.9% of DM for fine (1 mm) and coarse (4 mm), respectively. For sorghum, starch content was 72.1% and 71.4% of DM for fine (1 mm) and coarse (4 mm), respectively. Moreover, in vitro rumen starch degradability and rate of degradation were 78.1% and 22.5% h^−1^ (fine corn), 63.5% and 16.8 h^−1^ (coarse corn), 68.3% and 14.4 h^−1^ (fine sorghum), 60.1% and 13.1 h^−1^ (coarse sorghum).

**Table 3.** Particle size distribution and nominal geometric mean particle size (NGMPS) of corn and sorghum milled at 1- and 4-mm screen

Sieves,^1^ (µm)	Corn		Sorghum	
	Fine (1 mm)	Coarse (4 mm)	Fine (1 mm)	Coarse (4 mm)
**2,000**	—	4.49	—	3.01
**1,180**	0.160	24.64	0.28	29.68
**850**	0.687	16.21	0.98	25.68
**500**	18.97	14.89	18.47	17.86
**250**	34.21	14.67	32.97	10.94
**125**	32.02	14.99	31.77	6.39
**Pan**	13.96	10.11	15.50	6.45
**NGMPS** ^2^ **, µm**	255.55	550.14	250.62	728.54

^1^Particle size distribution is given as the proportion (%) of total material retained on each sieve.

^2^Nominal geometric mean particle size.

### Milk yield and composition

The effects of dietary SS and PS on milk yield and composition are presented in Table 4. No interaction was observed between SS and PS on milk yield and composition. Milk yield was not affected (*P* = 0.51) by SS; however, it was greater (*P* = 0.05) for cows fed fine grains than cows fed coarser grains (25.32 and 23.16 kg/d, respectively). Milk fat and milk protein were not affected (*P* > 0.05) by SS nor by PS. Content of milk lactose was greater (*P* = 0.05) in cows fed corn than sorghum diets, but the yield of lactose did not differ between SS. However, lactose yield was lower (*P* = 0.04) in cows fed coarsely than finely ground grains. The MUN was lower for cows fed corn compared to sorghum diets. The ECM was not affected by SS or PS (*P* = 0.43), but feed efficiency was affected by SS, with greater efficiency for cows fed sorghum diets than corn diets (*P* = 0.01).

**Table 4.** Dry matter intake (DMI), milk yield and milk composition of lactating dairy cows fed corn or sorghum varying in particle sizes

Item	Treatment				SEM	*P*-value		
	Corn		Sorghum					
	Finely	Coarse	Finely	Coarse		SS	PS	SS × PS
DMI, kg/d	25.5	24.8	22.6	21.4	1.48	0.03	0.45	0.83
MY, kg/d	25.3	23.8	25.4	22.5	2.26	0.51	0.05	0.45
BW, kg	723.0	706.1	720.7	692.8	27.67	0.49	0.08	0.62
DMI, % of BW	3.6	3.5	3.1	3.1	0.26	0.05	0.89	0.99
ECM,^1^ kg/d	27.9	27.3	28.9	26.2	2.29	0.99	0.22	0.43
Feed efficiency^2^	1.11	1.10	1.28	1.23	0.09	0.01	0.44	0.63
Milk fat								
%	4.02	4.34	4.33	4.52	0.25	0.34	0.32	0.79
kg/d	1.01	1.03	1.09	1.01	0.09	0.69	0.65	0.51
Milk protein								
%	3.58	3.60	3.56	3.58	0.07	0.74	0.70	0.98
kg/d	0.90	0.85	0.90	0.80	0.07	0.49	0.08	0.47
Milk lactose								
%	4.80	4.72	4.65	4.60	0.08	0.05	0.23	0.81
kg/d	1.22	1.13	1.19	1.04	0.12	0.24	0.04	0.55
MUN, mg/dL	14.36	14.89	16.99	17.09	0.86	0.01	0.41	0.58

^1^ECM (energy-corrected milk) = [0.327 × milk yield (kg)] + [12.95 × fat yield (kg)] + [7.2 × protein yield (kg)] (Orth, 1992).

^2^Feed efficiency = ECM/DMI.

^3^Significance of main effects of statistical model: source of starch in the diet (SS), particle size of the source of starch (PS), interaction of source of starch in the diet with particle size (SS × PS).

DMI, dry matter intake; MY, milk yield; MUN, milk urea nitrogen; BW, body weight.

### Ruminal pH and fermentation

**Figure 1.**
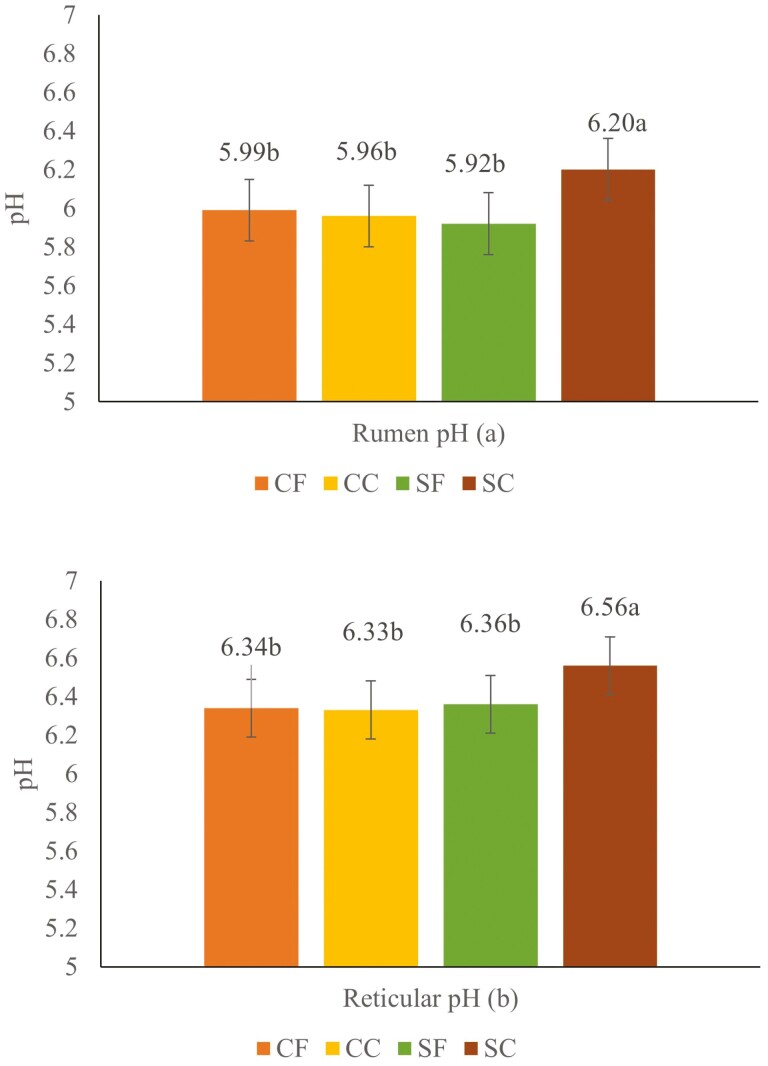
Effects of particle size (fine and coarse) and starch source (corn and sorghum) on rumen pH (a) and reticular pH (b); Means with different superscripts differ (*P* < 0.05); CF = finely ground corn; CC = coarsely ground corn; SF = finely ground sorghum; SC = coarsely ground sorghum.

**Figure 2.**
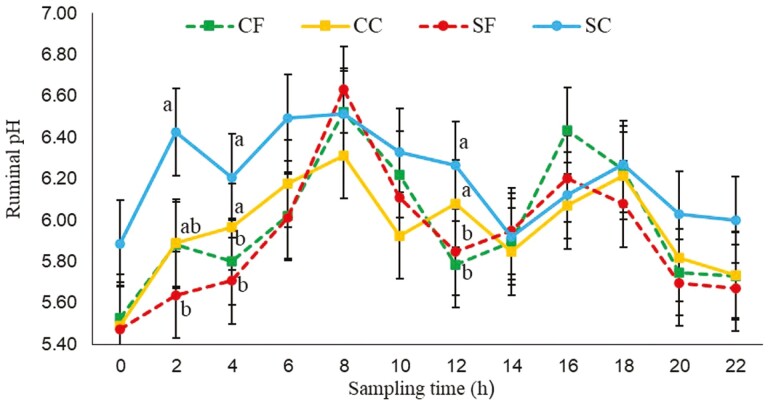
Effects of particle size (fine and coarse) of corn and sorghum diets on rumen pH over sampling time; CF = finely ground corn; CC = coarsely ground corn; SF = finely ground sorghum; SC = coarsely ground sorghum. Sampling time 0 = 3 h after feeding. Means with different superscripts differ (*P* < 0.05) for the interaction between particle size × time; The *P*-values were particle size = 0.0032; starch source = 0.044; time = <0.001; interaction particle size × source = 0.0006; interaction particle size × time = 0.04; interaction starch source × time = 0.98; interaction particle size × starch source × time = 0.92.

Starch source (*P* = 0.04), PS (*P* = 0.04), interaction between SS × PS (*P* < 0.01), and interaction between PS × time (*P* = 0.05) were significantly affected by the treatments. No effects were observed for the interactions between SS × time (*P* = 0.98) and the interaction among SS × PS × time (*P* = 0.92). For the interaction between SS × PS coarse sorghum had higher pH (6.20; Figure 1a) than fine sorghum (5.92), coarse corn (5.96), and fine corn (5.99). Regarding the interaction between PS × time, coarse particles had greater pH at 2, 4, and 12 h of sampling time compared to fine ­particles ­(Figure 2). For reticular pH no differences were observed for the interactions SS × time (*P* = 0.94), PS × time (*P* = 0.14), and SS × PS × time (*P* = 0.78). However, a significant effect was observed for SS (*P* = 0.02), PS (*P* = 0.01), and the interaction SS × PS (*P* = 0.03; Figure 1b).

There was an interaction between SS and PS on total VFA concentration (*P* = 0.01; Table 5). Finely grinding sorghum increased total VFA concentration compared to coarse sorghum; however, finely grinding corn did not affect VFA concentration.

**Table 5.** Ruminal NH_3_–N and VFA concentrations of lactating dairy cows fed corn or sorghum varying in particle size

Item	Treatment				SEM	*P*-value^1^		
	Corn		Sorghum					
	Finely	Coarse	Finely	Coarse		SS	PS	SS × PS
NH_3_–N, mg/dL	15.96	18.06	18.55	18.82	2.54	0.05	0.17	0.29
Total VFA, mM	112.05^ab^	115.90^a^	113.57^a^	108.34^b^	7.73	0.08	0.68	0.01
VFA molar proportions, mol/100 mol								
Acetate	62.80^b^	67.0^a^	65.84^ab^	64.07^ab^	5.36	0.97	0.28	0.01
Propionate	33.87	32.96	31.22	28.68	2.93	< 0.01	0.01	0.18
Acetate:propionate	1.91	2.10	2.21	2.33	0.21	< 0.01	< 0.01	0.39
Butyrate	8.88^bc^	9.15^ab^	9.47^a^	8.74^c^	0.62	0.46	0.07	< 0.01
Isobutyrate	2.39	2.51	2.63	2.65	0.43	0.01	0.20	0.36
Valerate	2.55	2.58	2.62	2.47	0.22	0.64	0.27	0.07
Isovalerate	1.57^c^	1.63^c^	1.80^a^	1.71^b^	0.16	< 0.01	0.62	0.01

^1^Significance of main effects of statistical model: source of starch in the diet (SS), particle size of the source of starch (PS), and interaction of source of starch in the diet with particle size (SS × PS).

No effect was observed for PS or interaction between SS and SP on the ruminal NH_3_-N concentration. However, ruminal NH_3_–N concentration (mg/dL) was lower for cows fed corn compared with cows fed sorghum diets (*P* = 0.05). There was an interaction between SS and PS on acetate, butyrate and isovalerate (*P* < 0.01) but not on propionate, acetate:propionate, and isobutyrate. Acetate concentration was lower for corn when finely ground, whereas, finely grinding sorghum did not affect acetate. The concentration of ruminal propionate was affected by SS and PS, with greater propionate observed in corn diets (*P* < 0.01) and finely ground diets (*P* < 0.01). However, butyrate and isovalerate were greater for finely ground sorghum than corn, as finely griding corn did not increase the levels of butyrate and isovalerate. Acetate to propionate ratios decreased for diets with corn and lower PS (*P* < 0.01). Lower ruminal concentrations of Isobutyrate was affected by SS, with lower content in the corn diet compared with sorghum diets (*P* < 0.01).

### Nutrient intake, flows, and digestibility

Treatment effects on intake, flow and digestibility of DM, OM, starch, and NDF are presented in Table 6. No interaction was observed between SS and PS on intake, flow to the reticulum and apparent digestibility of nutrients (*P* > 0.05). Compared to sorghum as a SS, cows had greater DM, OM, starch, and NDF intake when fed corn diets (*P* < 0.05). Intake of nutrients was affected by SS, with greater intake in cows fed corn diets compared with cows fed sorghum diets. The PS had no effect on intake of nutrients (*P* > 0.05).

**Table 6.** Intake, flow, and digestibility of nutrients in the lactating dairy cows fed corn or sorghum varying in particle sizes

Item	Treatment				SEM	*P*-value		
	Corn		Sorghum					
	Finely	Coarse	Finely	Coarse		SS	PS	SS × PS
DM								
Intake, kg/d	25.50	24.81	22.58	21.39	1.49	0.03	0.45	0.83
Apparent ruminal digestion, %	41.44	44.28	33.89	39.94	3.63	0.04	0.10	0.50
Flow at reticulum, kg/d	14.73	13.80	14.86	12.67	0.63	0.46	0.05	0.36
Apparent total-tract digestion, %	63.26	59.10	61.77	58.49	2.71	0.70	0.21	0.87
OM								
Intake, kg/d	23.70	23.06	20.97	19.83	1.37	0.02	0.42	0.82
Apparent ruminal digestion, %	49.81	52.97	44.47	50.45	3.37	0.14	0.09	0.56
Flow at reticulum, kg/d	11.70	10.82	11.63	9.71	0.59	0.36	0.06	0.41
Apparent total-tract digestion, %	64.91	60.37	63.65	59.54	2.70	0.71	0.16	0.94
Starch								
Intake, kg/d	6.77	6.50	5.68	5.26	0.50	0.02	0.19	0.76
Apparent ruminal digestion, %	88.77	83.76	85.59	83.10	1.55	0.15	0.02	0.32
Flow at reticulum, kg/d	0.76	1.04	0.82	0.88	0.09	0.39	0.02	0.08
Apparent total-tract digestion, %	96.29	87.84	92.21	86.93	2.15	0.29	0.02	0.49
NDF								
Intake, kg/d	7.84	7.86	6.76	6.58	0.52	0.04	0.88	0.85
Apparent ruminal digestion, %	28.32	33.18	32.14	36.57	2.15	0.07	0.03	0.90
Flow at reticulum, kg/d	5.59	5.26	4.64	4.18	0.42	0.05	0.37	0.88
Apparent total-tract digestion, %	35.15	37.65	39.29	41.84	2.35	0.04	0.15	0.99

^1^Significance of main effects of statistical model: source of starch in the diet (SS), particle size of the source of starch (PS), and interaction of source of starch in the diet with particle size (SS × PS).

DM, dry matter; OM, organic matter; NDF, Neutral detergent fiber.

Apparent ruminal digestion of DM was affected by SS (*P *= 0.04), with greater digestibility in cows fed corn diets compared to cows fed sorghum-based diets. The NDF ruminal digestibility tended to be greater in cows fed sorghum diet (*P* = 0.07). There was no effect of SS on OM and starch ruminal digestibility. Decreasing PS increased apparent digestibility of starch in the rumen (*P = *0.02) but decreased NDF digestibility in the rumen (*P *= 0.03). The digestibility of DM and OM in the rumen tended (*P *= 0.09) to be greater for cows fed coarsely ground diet. The flow of DM, OM, and starch was not affected by SS but that of NDF was greater in corn than sorghum-based diets (*P *= 0.05). Decreasing PS reduced the flow of starch to the reticulum (*P *= 0.02), increased the DM flow (*P *= 0.05) and tended to increase OM flow (*P *= 0.06) but PS had no effect on the flow of NDF to the reticulum.

The apparent total tract digestibility of starch was greater for finely ground diets than coarsely ground diets (*P* = 0.02), but it was not affected by SS. Conversely, apparent total-tract NDF digestibility (**TTNDFD**) was affected by SS (*P *= 0.04) but not by PS (*P* > 0.05). Cows fed corn diet had a lower TTNDFD than cows fed sorghum-based diet. The apparent total-tract digestibility of DM and OM were unaffected by SS or by PS (*P* > 0.10).

## Discussion

Processing had different effects on grains, resulting in different PS distribution and GMPS. Variation in PS can influence starch digestibility (Ferraretto et al., 2013; Gallo et al., 2016). Moreover, decreasing the mean particle length is expected to increase starch digestibility (Ferraretto et al., 2013) by increasing the surface area for bacterial and enzymatic digestion (Huntington, 1997). Overall, the chemical composition of corn and sorghum were within the expected ranges (Huntington, 1997; NRC, 2001), with higher starch content and lower NDF in corn than sorghum. The lower and slower digestibility of starch in sorghum may be due to the starch-protein matrix, which is more resistant to moisture, microbial attack and enzyme penetration (Rooney and Pflugfelder, 1986; Theurer, 1986; Kotarski et al., 1992). The presence of antinutritional factors such as polyphenols and phytate that may bind to amylase may also reduce its activity (Björck and Nyman, 1987).

Similarly to other studies, DMI was not affected by particle size (Rémond et al., 2004; Fredin et al., 2015; Brossillon et al., 2018). Apart from similar ruminal starch digestibility reported in this study, cows were fed diets formulated to contain similar starch concentrations, potentially supplying cows with the same energy levels. Therefore, the similarity in diets’ energy level and digestibility among treatments may explain the lack of effect of PS on DMI.

It is well documented that the DMI is influenced by several dietary factors, with dietary forage NDF content being the major factor affecting DMI in ruminants, by rumen fill (Mertens, 1987). The dietary NDF content of this study ranged from 30.8% to 32.1%, which may not have negative response on voluntary DMI due to ruminal distension (Allen, 2000). The present study is in line with Fredin et al. (2015) that also did not observe effects on DMI when the NDF concentration of the diets ranged from 26.9% to 32.1%.

It is important to note that this study had a small number of observations (*n* = 4) this is particularly important for milk production and composition; therefore, readers should interpret the data sensibly; however, the main goal of this study was to evaluate the effects of starch source, particle size, and their interaction on metabolism; milk production and composition were an extra set of information that help us tie together the metabolism data, future studies with greater animal numbers are warranted. Milk yield was affected by PS.

Inconsistencies have been reported in the literature with some studies reporting increased milk yield (Knowlton et al., 1996, 1998; Rémond et al., 2004) or unaffected milk yield (Callison et al., 2001; Fredin et al., 2015; Brossillon et al., 2018) milk yield. In a meta-analysis, Ferraretto et al. (2013) concluded that milk yield is unaffected by mean PS, contrary to a meta-analysis by Firkins et al. (2001), who reported that fine grinding increases milk production due to greater ruminal starch digestibility, which increases the concentration of VFA and ruminal bacterial yields, supporting milk synthesis (Yu et al., 1998).

Reports on the effects of PS on milk composition in the literature are inconsistent. For example, previous studies observed no effect of particle size on milk fat and protein (San Emeterio et al., 2000; Fredin et al., 2015; Ferraretto et al., 2013; Brossillon et al., 2018), opposing other studies (Knowlton et al., 1996; Firkins et al., 2001; Rémond et al., 2004). In the present study, we observed a tendency for increased milk protein and lactose content (kg/d) when cows were fed fine corn and sorghum, which was related to the increase of milk yield. Milk yield for cows fed fine diets was 2.2 kg greater than coarse diets, supporting greater component production. Several factors can affect milk composition, for example, milk protein percentage is highly correlated with rumen starch digestibility and negatively correlated to milk fat percentage (Theurer et al., 1999; Firkins et al., 2001). The increased fermentation of rumen starch has been demonstrated to have a positive effect on microbial protein synthesis, leading to an increase in protein synthesis in the mammary gland and subsequently increasing milk protein production (Firkins et al., 2001; Jenkins and McGuire, 2006; Rius et al., 2010). However, it is important to note that this process can also have a negative impact on milk fat production. Increased fermentable starch in the rumen results in greater levels of propionate, which stimulates gluconeogenic and insulin secretion. As a result, fatty acid mobilized from adipose tissue to the mammary gland is reduced, ultimately leading to a decrease in milk fat production (Bauman and Griinari, 2003). Moreover, with increasing ruminal starch digestibility milk fat content decreases as the ratio of acetate to propionate decreases, with increasing ruminal starch digestibility (Bauman and Griinari, 2001). It is important to emphasize that this process can also have positive effects on lactose yield synthesis and milk yield. Increases in propionate and glucose resulting from the increased fermentation have been found to support lactose synthesis and milk yield, ultimately leading to an overall increase in milk production. This may explain the 2.2 kg increase in milk yield observed in the present study. However, it is important to consider the low number of animals of the present study and the negative effect on milk fat production, as discussed earlier.

Decreased MUN for cows fed the corn diets could be attributed to a lower (1.73% units) dietary CP content of corn diets compared to the sorghum diets, or it could potentially indicate an improvement in ruminal nitrogen utilization in the corn diets. A greater feed efficiency for cows fed sorghum diets than corn diets could relate to lower DMI.

The decreased ruminal pH could be explained by increased ruminal starch digestibility with decreasing particle size, increasing VFA concentrations potentially resulting in lower pH (Fredin et al., 2015). Although the rumen and reticular pH fluctuated across sampling time with some ruminal pH below 5.8, the average ruminal pH for all treatments were above 5.8, which is considered as a threshold for rumen acidosis (Penner et al., 2007). The risk for subacute rumen acidosis is known to increase when ruminal pH drops below 5.8 for more than 5 to 6 h/d (Zebeli et al., 2012). An interaction (SS × PS) observed for rumen and reticular pH, indicates that the effects of decreasing particle size was more pronounced in sorghum. As mentioned earlier, coarse sorghum had higher pH than fine corn and sorghum, and coarse corn. This was likely due to lower surface area in the coarse sorghum that was lower compared to fine corn and sorghum, and coarse corn. Studies evaluating sorghum grain DM and starch ruminal digestibility have been show that intact sorghum grain has low ruminal digestibility. For example, McAllister et al. (1990) observed that only 19% of intact sorghum grain was degraded in the rumen after 120 h of incubation. Furthermore, observed an increase of 2.61-fold in effective ruminal starch disappearance for sorghum cut manually in four pieces compared to intact sorghum grains. The PS × time interaction implies that the effects on rumen and reticular pH depend on the time of fermentation. It is worth noting the increase in isovalerate and BCVFA with sorghum, which could indicate greater deamination during sorghum fermentation, increasing ruminal ammonia concentrations, as observed in this study. The decreased ruminal NH_3_–N concentration on corn diets could be attributed to increased ruminal starch digestibility thereby reducing the deamination process or improving the microbial capture of released AA or released NH_3_–N in the rumen. Starch fermentability affects VFA concentration, therefore, the greater concentration of propionate in cows fed fine and corn diets could be because of increased rumen fermentation or possibly a lower absorption as compared to coarse and sorghum diet. Starch in sorghum has a lower digestibility because of a strong starch-protein matrix (Herrera-Saldana et al., 1990; Kotarski et al., 1992). Decreased ruminal acetate in corn when finely ground decreased the ­acetate:propionate ratio, possibly because of increased concentration of propionate which is attributed to higher ruminal starch digestibility (Plascencia and Zinn, 1996).

The differences in starch intake could be attributed to DMI because starch and DM intake are positively correlated (Moharrery et al., 2014). However, in the present study the increase in starch intake did not result in increased milk yield. Independently of the SS cows had similar milk yield, this probably happened because cows were in late stage of lactation and both diets provided enough energy to supply the demand required for milk yield.

Ruminal starch digestibility can be highly variable (Moharrery et al., 2014). When evaluating starch digestion, it is important to consider source, for example corn, sorghum, and wheat, as well as site of measurement, such as omasal, reticular, or duodenal sampling. The ruminal digestibility values in our study were on the high range, independently of treatment; however, were within the expected values (72.0% to 89.9%) (Huntington, 1997; Fredin et al., 2015). Starch digestibility increases as PS decreases (Ferraretto et al., 2013). In agreement with our results, most previous studies reported increased TTSD for cows fed finely ground diets (Rémond et al., 2004; Fredin et al., 2015; Brossillon et al., 2018) because of its increased surface area, thereby facilitating ruminal microbes and enzymatic digestion compared with coarser particles (Huntington, 1997). Coarser particles decreased the amount of starch digested ruminally, and thereby increasing the amount of starch flowing to the small intestine. As a result, the total tract starch digestibility was reduced, suggesting that the decreased ruminal digestibility was not compensated for postruminally. Furthermore, in this study, diet with lower ruminal starch digestibility (coarse diets) showed a lower total tract digestibility compared to diets with greater starch digestibility (fine diets). This finding is consistent with other studies in the literature, which have shown a positive correlation between ruminal and total tract starch digestibility. For instance, Ferraretto et al. (2013) conducted a meta-analysis and found that increasing ruminal starch digestibility by 3.4% units could increase total tract starch digestibility by one percentage unit. Grain particle size is another factor that can affect both ruminal and total tract starch digestion, as suggested by (Owens et al., 1986). This may explain why the coarse diets in our study had lower ruminal and postruminal starch digestibility than fine diets.

The lower ruminal NDF digestibility of the corn and finely ground diets can be explained by the higher rumen starch digestibility, resulting in an unfavorable environment for cellulolytic bacteria growth and adherence thereby decreasing NDF digestibility (Firkins et al., 2001; Ferraretto et al., 2013). Even though cows fed fine corn and sorghum had decreased NDF digestibility, the increased in starch digestibility may have provided enough energy to the cows, especially considering their late stage of lactation, resulting in no differences in milk yield. This study supports that ruminal starch digestion negatively affects ruminal fiber digestion (Oba and Allen, 2003). The lack of SS × PS interaction for production, intake, flow, and ­digestibility of nutrients indicated that the effects of PS was observed regardless of the SS.

## Conclusion

In this study, no starch sources and particle size interactions were observed for production, intake, flow, and digestibility of nutrients. However, interactions were observed in ruminal and reticular pH, VFA concentrations and in some VFA molar proportions (acetate, butyrate, valerate, and isovalerate) but not on the ruminal NH_3_–N concentration. Ruminal and reticular pH were greater when sorghum was coarsely ground compared to coarsely ground corn, which also showed lower total VFA concentration. The acetate concentration was lower in corn when finely ground; however, no interaction was observed on propionate. Starch digestibility did not differ between starch sources, however, decreasing particle size increased ruminal digestibility of starch, reducing the flow of starch to the reticulum but decreased NDF digestibility in the rumen. Decreasing particle size increased apparent total tract starch digestibility. This study confirms that coarser particles can allow some of the starch digestion to be shifted from the rumen to the small intestine. It may, however, reduce total starch digestibility if ruminal digestion is not compensated for postruminally. The lack of SS × PS interaction for production, intake, flow, and digestibility of nutrients indicates that the effects of particle size was observed regardless of the starch source. However, the interactions between starch sources and particle sizes observed in pH and VFA concentrations indicates that the effects of decreasing particle size were more pronounced in sorghum. This study adds to the body of knowledge of starch source and particle size and can have practical implications, notably when precision feeding is gaining more attention.

## Abbreviations

ADF: acid detergent fiber
ADL: acid detergent lignin
BW: body weight
CP: crude protein
DMI: dry matter intake
FP: reticular fluid phase
GMPS: geometric distribution of particle sizes
LP: reticular large particle
MUN: milk urea nitrogen
MY: milk yield
NDF: neutral detergent fiber
NFC: nonfiber carbohydrate
NGMPS: nominal geometric mean
OM: organic matter
pdNDF: potentially digestible NDF
PS: particle sizes
RTD: reticular true digesta
SP: reticular small particle
SS: starch sources
TMR: total mixed ration
TTNDFD: total-tract NDF digestibility
uNDF: undigestible NDF
VFA: volatile fatty acids
